# HGF/MET and the Immune System: Relevance for Cancer Immunotherapy

**DOI:** 10.3390/ijms19113595

**Published:** 2018-11-14

**Authors:** Federica Papaccio, Carminia Maria Della Corte, Giuseppe Viscardi, Raimondo Di Liello, Giovanna Esposito, Francesca Sparano, Fortunato Ciardiello, Floriana Morgillo

**Affiliations:** Division of Medical Oncology, Department of Precision Medicine, School of Medicine, University of Campania “Luigi Vanvitelli”, Via Pansini n.5, 80131 Naples, Italy; carmy.dellacorte@gmail.com (C.M.D.C.); giuseppe.viscardi@hotmail.it (G.V.); diliello90@gmail.com (R.D.L.); espositogiovanna87@gmail.com (G.E.); franci.sparano@gmail.com (F.S.); fortunato.ciardiello@unicampania.it (F.C.)

**Keywords:** HGF, MET, immune system, cancer, immunotherapy

## Abstract

An overactivation of hepatocyte growth factor (HGF)/mesenchymal-epithelial transition factor (MET) axis promotes tumorigenesis and tumor progression in various cancer types. Research data recently evidenced that HGF/MET signaling is also involved also in the immune response, mainly modulating dendritic cells functions. In general, the pathway seems to play an immunosuppressive role, thus hypothesizing that it could constitute a mechanism of primary and acquired resistance to cancer immunotherapy. Recently, some approaches are being developed, including drug design and cell therapy to combine MET and programmed cell death receptor-1 (PD-1)/programmed cell death receptor-ligand 1 (PD-L1) inhibition. This approach could represent a new weapon in cancer therapy in the future.

## 1. Introduction

The *mesenchymal-epithelial transition factor* (*MET*) gene encodes for a membrane-bound receptor tyrosine kinase (RTK) that is mainly expressed in epithelial cells, the MET receptor. Physiologically, MET RTK is activated by a serum ligand, the hepatocyte growth factor (HGF), which is produced predominately by stromal cells and fibroblasts. MET activation triggers, through subsequent phosphorylations, various intracellular signaling cascades, including proliferation and survival pathways such as the extracellular signal-regulated kinase 1 and 2 (ERK1/2)/mitogen-activated protein kinases (MAPK) and the phosphoinositide 3-kinase (PI3K)/protein kinase B (AKT) and inflammation pathways such as signal transducer/activator of transcription (STAT) and nuclear-factor-κB (NF-κB) [1].

Physiologically, the HGF/MET pathway plays a main role in embryonic development, sustaining stem cell growth in early phases [2,3] and regulating the polarity and motility of more differentiated cells in later ones. The core of this process is the induction of the epithelial-to-mesenchymal (EMT) transition, which represents in organogenesis the gain of migratory abilities by multipotent cells that can differentiate into various cell types [4]. Similarly, HGF/MET signaling is involved in regeneration and tissue repairs [5,6].

An over-activation of the HGF/MET axis promotes tumorigenesis and tumor progression in various cancer types [7]. This happens through genomic activation, like germline MET mutation, such as in hereditary papillary renal cell carcinoma [8] or sporadic MET mutations, detected in various cancer types, including brain, gastric, and head and neck cancers [9] or even protein over-expression. In particular, MET protein levels increase in metaplasia-dysplasia-adenocarcinoma evolution in esophageal cancer [10], while higher levels of HGF are detectable during the carcinogenesis of gliomas [9], breast cancers, osteosarcomas, and melanomas [11,12].

Taken together, these findings elucidate a leading role of *MET* as a proto-oncogene in tumorigenesis.

Moreover, changing in *MET* expression explored in clinical settings revealed that it is a mediator of anti-cancer drugs resistance, like EGFR inhibitor resistance in non-small cell lung cancer (NSCLC) [13,14] and colorectal cancer (CRC) [15], and also that it correlates with worse prognosis and aggressiveness [16], such as in hepatocellular carcinoma [17], breast cancer [18], and CRC [19].

**Figure 1:**
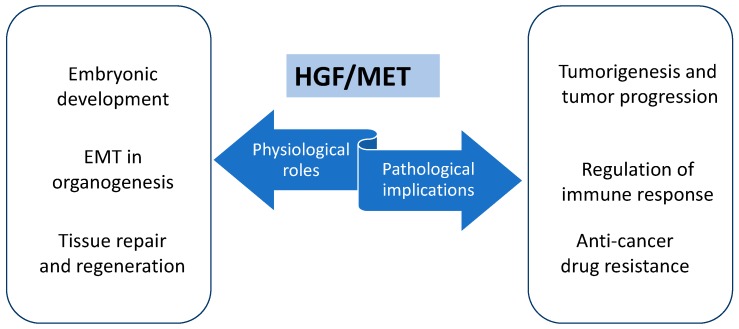
Roles of the hepatocyte growth factor (HGF)/mesenchymal-epithelial transition factor (MET) pathway.

MET role as a marker of resistance to anti-tumor therapy and bad prognosis mainly relies on its ability in inducing EMT, and consequent changes in gene and protein expression phenotype in cancer cells, which become more malignant and highly invasive [20]. In the last few decades, researchers have pointed out that oncogene alterations are involved in cancer progression, and they are potentially targetable, with new molecularly targeted agents. Even if these agents are selective and able to arrest tumor growth, the majority of patients sooner or later experience relapse. Resistance is the result of complex interactions among various receptor tyrosine kinases (RTKs) and other proliferative signals, including MET, and they have been the object of previous works of our group [20,21] (Figure 1).

## 2. Relevant Immune Pathways for Cancer Treatment

Among the main so-called hallmarks of cancer identified by Hanahan and Weinberg [22], there the “immune surveillance”: the role played by the immune system against the formation and progression of incipient neoplasia, late-stage tumors, and micro-metastases. However, immune surveillance is counteracted by the ability of cancer cells to escape recognition and killing by immune cells. Indeed, there is a constant dynamic interaction between immune cells and cancer cells: for example, cancer cells may block infiltrating T-cells and natural killer (NK) cells by secreting TGF-β or other immunosuppressive factors [23]. Another mechanism is the recruitment of immunosuppressive cells such as regulatory T-cells (Tregs) and myeloid-derived suppressor cells (MDSCs) [24,25]. Thus, “immunoevasion” is another key hallmark of cancer [22]. Several target proteins acting as key checkpoints in the regulation of immune cell activity have been described. One of the earliest to be identified was the cytotoxic T lymphocyte associated antigen-4 (CTLA-4). The expression of a CTLA-4 molecule is upregulated after the activation of T-cells [26]. CTLA-4 induces T-cell cycle arrest [27] and competitively binds with B7 molecules present on the antigen presenting cell (APC), thereby inhibiting T-cell activity stimulation [28]. Programmed cell death receptor-1 (PD-1) is another important molecule that is expressed by activated T-lymphocytes. Upon binding with programmed cell death receptor-ligand 1 (PD-L1), it downregulates T-cell activity and proliferation [29]. More recently, other checkpoints have been identified that deliver a positive costimulatory signal, such as the 4-1BB receptor (CD137) and the OX40 molecule [30,31,32].

In particular, PD-L1 is an extracellular protein that downregulates immune response primarily in peripheral tissues through binding to its two receptors, PD-1 and B7.1. PD-1 is an inhibitory receptor expressed on T-cells following T-cell activation, which is sustained in states of chronic stimulation, such as in chronic infection or cancer [33,34]. Their interaction delivers an inhibitory signal on T-cell proliferation, cytokine production, and cytolytic activity, leading to the functional inactivation or exhaustion of T-cells. B7.1 is a molecule that is expressed on APCs and activated T-cells. PD-L1 can also bind to B7.1 on T-cells, and APCs can mediate the downregulation of immune responses, including the inhibition of T-cell activation and cytokine production [35].

The overexpression of PD-L1 on cancer cells has been reported to block anti-tumor immunity, resulting in immune escape [33], and blocking the PD-1/PD-L1 pathway is a key strategy for restoring tumor-specific T-cell immunity.

## 3. HGF/MET and Immune System in Cancer

Research data has demonstrated that HGF/MET signaling is also involved in immune responses [36,37,38]; nevertheless, its role is still unclear: it can act as an immunosuppressive stimulus by negatively affecting dendritic cells (DC) and T lymphocytes, or as an immune-positive stimulus, by promoting the recruitment of DC [39,40,41], B cells [42], and T lymphocytes [43,44].

HGF itself is also able to control the migration of B and T lymphocytes, and to counteract the anti-inflammatory effect of TGF-β [45,46]; as an example, in experimental animal models of auto-inflammatory diseases, higher levels of HGF have a defensive role against inflammation and fibrosis [47,48].

Moreover, HGF itself induces the expansion of all blood cell types precursors, in cooperation with other hematopoietic stimuli [49].

Specifically, in the context of cancer, the role of HGF/MET is very complex: HGF is secreted by cancer cells themselves, and also by stromal cells. Its oncogenic and pro-metastatic effects are well known and studied, while very recently, the interest in its effect on the immune system in cancer is increasing, due to the direct therapeutic implications in the clinical scenario of new immunotherapy drugs [50].

First of all, MET itself can be recognized as a tumor-associated antigen (TAA) by CD8 cytotoxic T-cells, and this mechanism can trigger immune system activation against cancer cells that overexpress MET [51].

Until now, the most widely investigated role of MET is that which is played on DCs. These are involved in the presentation of TAAs, and they induce the activation of regulatory T-cells (CD4^+^) that control cytotoxic CD8^+^ T-cells. It has been shown that HGF is able to increase this function, thus suggesting a positive role in anti-cancer immunity [39,47,52,53]. However, some papers have demonstrated that HGF can also be a potent negative regulator of DC function, and they can induce an increase of T regulatory lymphocytes, a decrease of interleukin-17-producing lymphocytes [39], and an increase in interleukin-10 (IL-10) and transforming growth factor beta (TGF-β), known for their immunosuppressive role [40]. Thus, the inhibition of DCs, the decrease of CD8^+^ T lymphocytes, and the increase of T reg cells result in the inhibition of the immune response [39]. This inhibitory effect has also been demonstrated in monocytes, particularly with an induction towards a differentiation into regulatory IL-10 producing cells [54]. 

Besides antigen-presenting cells, the HGF/MET interplay with immune system is also identifiable in granulocytes. Interestingly, MET deletion in neutrophils enhances tumor growth and metastasis, as MET is required for chemoattraction and neutrophil-mediated cytotoxicity. In clinical samples, this phenomenon is represented by a correlation between MET deletion and reduced neutrophil infiltration to both primary tumor and distant metastases. In particular, tumor-derived TNF-α or other inflammatory stimuli can induce MET in human neutrophils, thereby leading to neutrophils transmigration across an activated endothelium and free radical production, which can actually induce cancer cell killing. This is an important issue to be considered when treating cancer patients with MET-inhibitors. Indeed, this mechanism could represent an escape from tumor killing upon MET-inhibitor treatment, as they also block the kinase activity in neutrophils [55]. This is a perfect example of the complex role that is played by MET in cancer. Indeed, if in some cases, HGF/MET is essential for cancer cell survival and it is one of the main drivers, in other cases it promotes anti-cancer effects. This would be of great importance when testing new combinations.

Another key topic is represented by the interaction of MET with other pathways and with immune check-points. Recently, it has been demonstrated that renal cancer cells, after they have been stimulated with HGF, display PD-L1 upregulation and co-localization with MET, and that PD-L1 upregulation was dependent on the PI3K pathway [56].

Moreover, the PI3K pathway is involved also in the HGF-mediated inhibition of DC [57], where is causes the inhibition of the NF-kB pathway at different levels, through mTOR [57], c-Src [57], or Bruton’s tyrosine kinase (Btk) [58]. 

**Figure 2:**
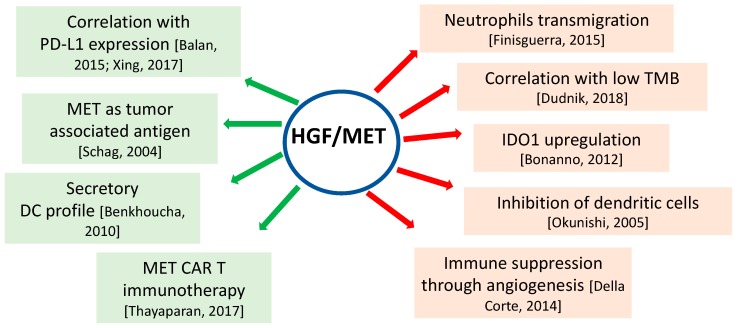
Multiple roles of HGF/MET in cancer immunotherapy. MET CAR T: MET chimeric antigen receptor T cell; IDO1: indoleamine 2,3-dioxygenase 1.

Apart from these preclinical data, there are also some clinical evidences. As an example, in multiple myeloma, HGF expression is related to a worse immune response deficiency through the upregulation of indoleamine 2,3-dioxygenase 1 (IDO1) [59], and this could be an important issue to consider, giving the role of PD-1 and PD-L1 in multiple myeloma immune evasion and progression [60] (Figure 2).

## 4. Implications of HGF/MET in Anti-Cancer Immunotherap

*MET* overexpression is very common in several types of solid cancers, and there are numerous experimental and clinical evidences of its role in mediating resistance to conventional anti-tumor therapies, thus making MET a very attractive druggable target [61]. *MET* overexpression is also often a negative prognostic factor itself, due to its intrinsic ability to induce cell cycle progression, cell migration, invasion, EMT, and angiogenesis [20]. Currently, three main strategies target MET kinase activity: the blockade of extracellular MET–HGF interaction by monoclonal antibodies directed against MET, and the inhibition of phosphorylation of the tyrosine kinase domain by small-molecule inhibitors or downstream inhibitors.

Moreover, in EGFR-driven lung cancer and CRC, MET activation is a very common cause of resistance to anti-EGFR drugs [14,15,20,62], so that the use of MET inhibitors could possibly overcome this resistance. Actually, in multiple MET-targeted therapies, both monoclonal antibodies and receptor tyrosine kinases, have been tested, with good results in preclinical models. Unfortunately, all of them have failed in clinical trials, possibly because studies were held in an unselected population [63,64,65]. For example, onartuzumab and rilotumumab, two anti-MET monoclonal antibodies, failed respectively in TNBC [66], and even in MET-positive gastric cancer [67].

In the last few years, the clinical scenario of anti-cancer therapies has seen a dramatic change with the introduction of immunotherapy agents. Immunotherapy is a kind of biologic cancer treatment that boosts the natural immune defenses of the patient to fight tumor cells. This includes monoclonal antibodies against negative immune checkpoints molecules, oncolytic virus therapy, T-cell therapy, and cancer vaccines [68]. Immune checkpoints are among the main proteins that mediate the interaction between cancer cells and the immune system. The PD-1 (programmed cell death-1) receptor/PD-L1 (programmed cell death ligand-1) is the best investigated pathway that is involved in cancer induced immune-suppression [69]. PD-1 is expressed on activated T-cells, and its ligands PD-L1 and PD-L2 are expressed on the surface of dendritic cells or macrophages, and they are all co-inhibitory proteins of the T-cell response. PD-1 activation by the PD-L1 expressed by cancer cells is a mechanism of the adaptive immune resistance of cancer cells, and it currently represents the main target of immunotherapy in cancer [69]. In the last few years, anti-PD-1/PD-L1 agents have been approved worldwide for the treatment of melanoma, lung cancer, urothelial cancer, and lymphoma [70,71,72,73]. Like any other type of cancer treatment, patients can be intrinsically resistant, or develop, at some point of the therapy, acquired resistance to immunotherapy, but mechanisms of resistance are still unknown.

In lung cancer, PD-L1 expression on tumor tissue, defined by immunohistochemistry and tumor mutational burden, based on whole exome sequencing analysis, are the only two positive biomarkers for the response to anti PD-1/PD-L1 immunotherapy, while variants in EGFR and STK11 genes were found to be associated, with no benefit in NSCLC patients [74,75,76]. Among the entire population of NSCLC, there is a small subgroup of patients harboring a *MET* exon 14 mutation (about 4%). These patients may benefit from MET-targeted therapy, as already anticipated by data from an expansion cohort of the phase I study of the MET-ALK inhibitor crizotinib (PROFILE 1001) [77]; other ongoing clinical trials are still evaluating the efficacy of more specific MET-inhibitors such as tepotinib (NCT02864992) in advanced NSCLC with *MET* exon 14-skipping alterations or *MET* amplification. A very interesting study by Sabari et al. retrospectively investigated the effectiveness of immune checkpoints inhibitors in *MET* exon 14-mutated NSCLC patients, and showed that, despite them expressing high PD-L1 levels, treatment responses were very rare, with very short durations of response and progression-free survival, and they were also lower than those observed with MET-targeted therapy [78]. Interestingly, *MET* exon14-mutated NSCLC patients showed very low TMB levels, similarly to BRAF and ROS1 mutant NSCLC, which represent other sub-groups of patients with an absence of clinical benefits from immunotherapy [79]. In our opinion, these data encourage the testing of combination strategies such as immunotherapy plus MET-inhibitors, plus/minus chemotherapy in this cohort of patients. In a similar study conducted on a gastric cancers dataset, Xing et al. correlated PDL-1 and PDL-2 expression to MET, and they found that the majority of tumors with high PDL-1/2 expression were MET-positive [80]. It is becoming more and more evident that PDL-1 is not the only marker to consider for guiding the selection of patients for immunotherapy, especially in the presence of oncogene alterations, and that MET expression/gene alterations are probably implicated in the resistance to single agent anti-PD-1/PD-L1 drugs. As is arguable from previously illustrated evidence, there are many factors that mediate this resistance: we can hypothesize that MET induces the migration of neutrophils from the bone marrow to the lymph nodes, where they can inhibit T-cell expansion [81]. Thus, a pharmacological inhibition of MET can potentially synergize with immunotherapy by avoiding this neutrophil mediated immune suppressive effect. Glodde et al. [81] demonstrated in immune competent mice that the addition of MET inhibitors to immunotherapy increases the numbers of active T-cells and also changes their phenotype, by reducing the proportion of exhausted T-cells. These results were independent from MET expression in the tumor models used, further suggesting that MET inhibition can have a role in increasing immunotherapy efficacies, not only in MET-driven tumors. Moreover, Kumai et al. [82] showed that MET expression itself behaves as a tumor-associated antigen and that it is able to activate CD4^+^ T-cells and to induce tumor cell killing in NK/T-cell lymphoma (NKTCL) cell lines. In particular, in this model, MET elicited a specific anti-tumor immune response, with three novel identified MET-induced T-cells epitopes. The activation of T-cells was stronger in the presence of MET-inhibitors, since they caused a reduction of the synthesis of TGF-beta, which is immune-suppressive, from tumor cells. Additionally, the presentation of MET-derived peptides by major histocompatibility complex class II (MHCII) to CD4^+^ T-cells was influenced by chaperon processing and autophagy, thus proposing an innovative potential role of autophagy inducers as immune activators. Finally, since HGF/MET stimulation increases the proliferation of NKTCL cells in vitro, MET inhibition again displayed a dual role: direct tumor killing for MET-dependent cell survival, and anti-tumor immune activation [82].

Recently, some approaches are under development, including drug design and cell therapy. A novel dual inhibitor of MET and PD-1 was designed by Sun et al. [83] and tested in multiple cancer cell-type models. It demonstrated a strong anti-proliferative and anti-metastatic effect in vitro and in vivo, and it reduced the production of inflammatory chemokines such as IL-6 and TNF-α, thus suggesting an important therapeutic potential, although still in the preclinical model stage [83].

Another MET-targeted immunotherapy approach that has already been tested in preclinical mesothelioma models is the MET chimeric antigen receptor (CAR) T-cell immunotherapy [84]. CAR T-cell therapy is an innovative treatment consisting of the genetic modification of patient’s T-cells that makes them able to kill specific cancer cells. In particular, T-cells are taken from patients’ blood, modified in the laboratory by inserting a specific CAR, and then re-injected into the patient. CAR-T therapy is one of the newest immunotherapeutic approaches to be introduced into clinical practice: this year, two CD19-specific CAR T-cell therapies have been approved by the Food and Drug Administration (FDA) for treatments of hematological cancers [85]. The efficacy of CAR T-cell therapies is very promising, but there are still many unaddressed safety concerns, especially in terms of long-term effects. For any human-derived therapy, FDA approval is based on preclinical and clinical efficacy, but also on the successful completion of safety processes for testing the infective, genetic, and purity characteristics of the products, both in vitro and in vivo [86]. With regard to EMA requirements, complete and updated references can be found at the following link: European Medicine Agency et the URL https://www.ema.europa.eu/human-regulatory/research-development/scientific-guidelines/multidisciplinary/multidisciplinary-cell-therapy-tissue-engineering (accessed on 12 November 2018).

In the study of Thayaparan et al., T-cells have been engineered to express HGF as a chimeric antigen to target MET-expressing cancer cells [84]. The efficacy of this approach was confirmed both in vitro and in vivo: a consistent level of cancer cell killing and tumor regression was detected in all models, and it was also accompanied by the release of IFN-gamma and IL-2 from MET-targeted CAR-T-cells, confirming that cancer cell death was immune-mediated.

Previously, Frigault et al. engineered novel CARs coding for immune signals molecules, such as CD28, ICOS, and 4-1BB, by using common cancer genes including MET as a referral to design the promoters to be transfected in T-cells [87].

Only preliminary data are available for MET CAR T on human samples: they come from the data of intra-tumor injection of MET CAR T-cells in breast cancer tumors. After injection, these tumors displayed necrosis, loss of MET, and infiltration of macrophages, which are markers of inflammation [88].

These data confirm the good activity of the CAR T method, and suggest subsequent evaluations in other cancer types with a significant MET expression, thus introducing a very innovative therapeutic potential of MET targeting.

## 5. Future Perspectives and Conclusions

From clinical datasets, only few datasets are already available on the resistance to the currently used anti-PD-1/PD-L1 immunotherapy, and further studies are needed to select and identify subpopulations of patients that can derive clinically consistent benefit from these drugs.

The HGF/MET pathway plays many distinct roles with an impact on tumor aggressiveness. Among these, a poorly explored one is that on the immune system. Considering the previously cited references, it is evident that this role is not sufficiently understood. Indeed, currently available data are quite contradictory, at least in some circumstances. For example, IDO1 upregulation appears to favor tumor growth [59], while the fact that MET itself can act as a TAA may enforce the immune system against the tumor [51]. Nevertheless, HGF/MET seems to have a preponderant immune-suppressive role in cancer immune response, through the direct inhibition of DCs and an indirect inhibition of active T-cell proliferation as mediated by neutrophils. Since immunotherapy agents in cancer have re-modeled the prognosis and the treatment of major cancer types in last years, future research on the MET role in the immune system are strongly encouraged. Moreover, giving the role of HGF/MET in inducing angiogenesis, and with the fact that pro-angiogenetic signals are themselves immunosuppressive, we think that deeper studies are needed to clarify whether there is a direct link between HGF/MET-induced vascularization and its autocrine signals, and the immune suppression of the tumor microenvironment in HGF/MET-positive tumors.

Various preclinical data support the immunological mechanisms and the potential efficacy of combining MET-inhibitors with immunotherapy; in a clinical context, dose-limiting toxicities could be an issue for this combination, and no data are currently available on their safety in humans. As a consequence, we can only make hypotheses on the probable side effects. Besides class-effect adverse events of MET targeting agents (peripheral edemas for instance) [64,65], HGF/MET proper immune effects are to be motifs of concern. In particular, the immunological targeting of MET may cause disequilibrium to the same immune response regulated by MET; thus, further clinical evaluation is strongly necessary. Major attention even needs to be played when combining MET inhibition with anti-PD1/PD-L1; for instance, since immunological disruption might be even stronger. The onset of very innovative immunotherapy approaches, like the engineered CAR-T method, is opening a new road toward more targeted immunotherapies. Results are promising from various cancer types, but also in this case, off targets effects should be carefully considered. CAR-T cell therapy, indeed, is accompanied by some important (even life-threatening) side effects, such as massive cytokine release syndrome [89], which could possibly be even more pronounced when used to target MET-expressing cells. Moreover, giving the multiple effects of HGF/MET inhibitors on angiogenesis, it can be argued that a deeper level of homeostasis can be perturbed with, as an example, vasculature impairments and worsening of the previous cited syndrome.

Another issue to be considered is the selectivity of action of the putative combination (anti-MET + anti-PD-1/PD-L1 or MET-CAR-T-cells). Indeed, the MET receptor is expressed with a plethora of normal cells, which implies the possibility of safety concerns to the effect on normal besides tumor cells. Too little data are now available; thus, further studies should help to quantify the off-target effects of these therapies. 

Other interesting approaches rely on the possibility of improving immune checkpoint inhibitor delivery through the use of nanotechnologies [90,91].

In conclusion, the HGF/MET pathway is one of the most important pro-oncogenic, pro-angiogenetic, and pro-metastatic signals in various cancer types. Interestingly, from various studies, its activation emerges as one of the main mediators of resistance to anti-cancer therapies, including the novel anti-PD-1/PD-L1 immunotherapies, thus reinforcing the potential of MET-targeted therapies and giving the rationale for testing them in combinational strategies or integrated innovative approaches, like CAR-T therapy, in multiple cancer types.
